# Machine Learning Predicts the Oxidative Stress Subtypes Provide an Innovative Insight into Colorectal Cancer

**DOI:** 10.1155/2023/1737501

**Published:** 2023-04-21

**Authors:** Haitao Zhong, Le Yang, Qingshang Zeng, Weidong Chen, Haibo Zhao, Linlin Wu, Lei Qin, Qing-Qing Yu

**Affiliations:** ^1^Jining First People's Hospital, Jining Medical University, Jining 272000, China; ^2^Shanghai Tianyou Hospital, Tongji University, Shanghai 200333, China; ^3^Department of Oncology, Tengzhou Central People's Hospital Affiliated to Jining Medical College, Tengzhou 277500, China

## Abstract

So far, it has been reached the academic consensus that the molecular subtypes are via genomic heterogeneity and immune infiltration patterns. Considering that oxidative stress (OS) is involved in tumorigenesis and prognosis prediction, we propose an innovative classification of colorectal cancer- (CRC-) OS subtypes. We obtain three datasets from The Cancer Genome Atlas Program (TCGA) and Gene Expression Omnibus (GEO) online databases. 1399 OS-related genes were selected from the GeneCards database. We remove the batch effect before conducting differentially expressed genes (DEGs) analyses between normal and tumor samples. Nonnegative matrix factorization (NMF) was used to perform an unsupervised cluster. Lasso regression and Cox regression were used to construct the signature model. DEGs, robust rank aggregation, and protein-protein interaction networks were used to select hub genes, and then use hub genes to predict OS subtypes by random forest algorithms. NMF identifies two OS-related subtypes of CRC patients. Eight OS-related gene signatures were built to predict the outcome of patients, based on the DEGs between two subtypes. A total of 61 DEGs overlap each dataset, and the RRA analysis shows that 17 genes are important in these three datasets, and 15 genes are shared genes between the two methods. PPI network suggests that five hub genes are confirmed, they are SPP1, SERPINE1, CAV1, PDGFRB, and PLAU. These five hub genes could predict the OS-related subtype of CRC accurately with AUC equal to 0.771. In our study, we identify two OS-related subtypes, which will provide an innovative insight into colorectal cancer.

## 1. Introduction

Nowadays, with an estimated 1,800,000 new cases and 900,000 deaths annually [1, 2], colorectal cancer (CRC) become the third most common cancer and the second leading cause of cancer death [3]. Despite rapid development in the diagnosis and treatments of CRC, the mortality remains high, especially in advanced stage at first diagnosis [4]. Therefore, the lack of biomarkers for early screening and prognosis prediction is still an urgent clinical problem to improve the treatments efficacy and reduce the cases mortality of CRC.

With the numerous studies on hallmarks of cancer, the characteristics of genomic variation in CRC have unique clonal, stromal, and immune dependencies [5]. So far, a molecular classification of CRC has been reached the academic consensus than the four molecular subtype groups via the current best description of the genomic heterogeneity [6]. In addition to the transcriptomic subtypes of CRC, the expression profile analysis of CRC showed that the immune infiltration patterns with different immune-tolerant microenvironment resulted in different effects of special immunotherapy [7, 8]. However, it is more notable that to maintain the high proliferation rate tumor cells, it demands high ROS concentrations, which the regulation of oxidative stress (OS) includes oxidative metabolism, for example, the conversion of the glycolytic pathway into the pentose phosphate pathway [9, 10]. And the prognosis of radiotherapy and chemotherapy treatments is influenced through OS modulation indicating that the OS is also highly significant for cancer therapy [11–14]. Therefore, we propose an innovative classification of CRC-OS subtypes, which has never been studied in-depth.

Machine learning is a computer technology applied artificial intelligence which has a widespread application in improving medical research and clinical decision in clinic such as diagnostics, precision medicine, and clinical trials of cancer. In our study, we aimed at identifying potential OS molecular subtypes and predicting CRC outcomes through the gene expression profile in multiple datasets. By utilizing the nonnegative matrix factorization (NMF) clustering algorithm, 350 OS-related differentially expressed genes (DEGs) were distinctly classified into two molecular subtypes (named C1 and C2) in three CRC cohorts. Among them, C1 was associated with a better prognosis. Moreover, based on the intersection of C1 and C2 DEGs, we established a novel OS-related prognostic signature by multivariate Cox regression model and validated its significant prognostic values for CRC patients. Finally, we also explored the hub genes for predicting OS subtypes in CRC.

## 2. Materials and Methods

### 2.1. Data Obtain

We obtain datasets from three individual datasets, colon adenocarcinoma (COAD) from The Cancer Genome Atlas Program (TCGA), including 41 normal samples and 473 tumor samples; GSE39582, including 19 normal samples and 443 tumor samples; GSE29621, including 65 tumor samples from Gene Expression Omnibus (GEO), respectively. Gene expression profile and clinic information of the above samples were downloaded by R package TCGA biolinks [15]. Oxidative stress- (OS-) related genes were checked by the GeneCards database (https://www.genecards.org/) and 1399 OS-related genes were selected for future analysis.

### 2.2. Batch Effect Correction

Because the above datasets resource from different individual databases, we use R package sva to reduce the batch effect between samples. We merge these three datasets and calculate the overlap genes, then use the combat method to remove the batch effect.

### 2.3. OS-Related Genes' Different Expressions

We extract 1399 OS-related genes from the above merge expression matrix which has been removed batch effect and then use the limma package to conduct differential expression analysis between normal and tumor samples. Absolute value of LogFC was set to more than 1, and the *p* value was set to less than 0.05.

### 2.4. Nonnegative Matrix Factorization Identify OS-Related Subtype

Nonnegative matrix factorization (NMF) is an excellent unsupervised learning algorithm that could identify the probable subtype between large samples. Here, we also use this method and aim to identify candidate OS-related subtypes in CRC samples. We set the rank from 2 to 10 using a method called brunet, and other algorithm parameters were set as default.

### 2.5. Subtypes Validate and Survival Analysis

After identifying candidate subtypes, we also use heat map and principal component analysis (PCA) to validate the results of the typing results. Survival analysis is also used to compare different subgroups. The survival difference was calculated by a log-rank test.

### 2.6. Different Expression Genes between Subtypes

To explore the potential mechanisms between subtypes and build a useful prediction model, we use the limma package to conduct differential expression analysis between groups. Here, we set the absolute value of LogFC as more than 0.5, and the *p* value also was set to less than 0.05.

### 2.7. Lasso Regression and Cox Regression to Identify Model Genes

A useful machine learning method, Lasso regression and Cox regression model were used to dimensionalize the data. We use the batch univariate Cox regression model to obtain the prognosis OS-related genes, and then we input the above results into Lasso regression and calculate the minimum value of lambda to get the important genes of the model. Next, the important genes of Lasso regression results will perform the last step, and multivariate Cox regression will conduct this procedure to select the candidate model genes.

### 2.8. A Signature Model Constructs to Predict the Overall Survival of CRC Patients

The multivariate regression model has selected candidate genes, which will be constructed as a signature. This signature was constructed in two steps, first, each candidate gene coefficient needs extract from multivariate regression results, and second, we calculate the risk score of each patient according to the following formula. Risk score = expression × gene A + expression × gene B + expression × gene C. After calculating all patients' risk scores, they will be divided into two groups, according to the median value. A survival curve will be used to demonstrate the differences between groups, and receiver operating characteristic (ROC) was used to evaluate the signature prediction ability.

### 2.9. DEGs Combine Robust Rank Aggregation to Identify Important DEGs

We calculate the different genes in each dataset and merge three DEGs results, then, we also use robust rank aggregation (RRA) method to select the important DEGs, and finally, we combine DEGs overlap results and RRA results to get the final overlap genes which are considered as important genes with different expression between subtypes. To explore the candidate mechanism of DEGs between subtypes, Kobas (http://kobas.cbi.pku.edu.cn/) was used to perform the enrichment analysis including Gene Oncology and KEGG analysis.

### 2.10. Protein-Protein Interaction Networks Analysis and Hub Gene Screened

We use the STRING database (https://cn.string-db.org/), which is an online search for known protein interactions to conduct protein-protein interaction networks (PPI) to show the internal interaction in important genes and use MCODE plugins, which are resources from Cytoscape. To identify the hub protein network, genes located in this network were considered hub genes.

### 2.11. Hub Gene Predicts OS Subtypes by Random Forest Algorithms

We perform hub genes to predict the OS subtype of CRC by random forest (RF) algorithms, which are included in the caret package. The detailed steps are listed here. The dataset of standardization, which has removed the batch effect, will be divided into two random datasets: one account for 70% as a training set and another is 30%, as a test set. Then, we use hub genes to predict the OS subtype of CRC in the training set and validate the prediction ability of these hub genes in the test set. Finally, we visualize the decision tree of the model. In this step, the most important is that all input gene expression needs to conduct min-max normalization, which will transfer gene expression values from 0 to 1.

## 3. Results

### 3.1. OS-Related Different Expressions of Genes

**Figure 1:**
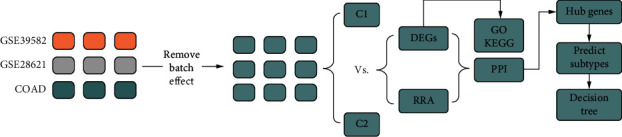
Workflow of the study. Three datasets (COAD, GSE39583, and GSE 28621) were obtained from TCGA and GEO including 1399 OS-related genes selected from the GeneCards database. Before DEGs analyses, the batch effect was removed. And nonnegative matrix factorization (NMF) was used to perform an unsupervised cluster. Hub genes were selected by Lasso regression and Cox regression to construct the signature model. DEGs, robust rank aggregation, protein-protein interaction networks were used to select hub genes to predict OS subtypes by random forest algorithms.

**Figure 2:**
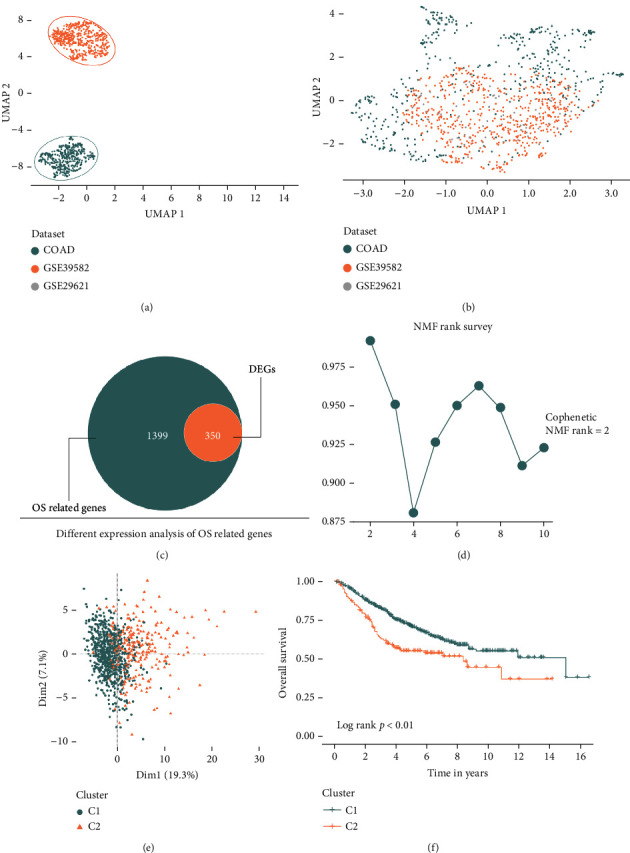
NMF cluster samples into two oxidative stress subtypes. Before batch correction, three datasets are scattered (a). After batch correction, the component data is evenly distributed (b). The OS-related DEGs are selected by OS-related genes and DEGs (c). *K* = 2 was best cut off by NMF analysis (d). PCA results show that two subtypes are grouped distinctly (e). Survival analysis demonstrates C2 patients with a poor prognosis, compared with C1 patients (f).

This study workflow is shown in Figure 1, and a total of 1041 samples were enrolled in our study, including 60 normal samples and 981 tumor samples, respectively. 1399 OS-related genes are selected from GeneCards, and we performed the remove batch effect before extracted this OS-related gene from 981 tumor samples. Before batch removal, we could find that samples are distributed in three different spaces (Figure 2(a)), and after removing the batch effect, all samples are distributed on average (Figure 2(b)).

### 3.2. Two OS-Related Subtypes of CRC Patients and DEGs

Some OS-related genes were expressed differently between normal and tumor tissues in CRC patients, so we conduct DEGs analysis to filter the above genes. These results show that 350 DEGs are selected, including 204 upregulated genes and 146 downregulated genes, respectively (Figure 2(c)). NMF conducts unsupervised clustering by these 350 DEGs. When rank = 2, the clustering result is best, and samples will be divided into two groups (Figure 2(d) and Supplement Figure 1). The PCA results also demonstrate the above conclusion (Figure 2(e)). Survival analysis results show that when compared with cluster 1, cluster 2 patients will obtain a poor prognosis (log-rank *p* < 0.01) (Figure 2(f)).

### 3.3. Eight OS-Related Gene Signatures Predict the Outcome of Patients

**Figure 3:**
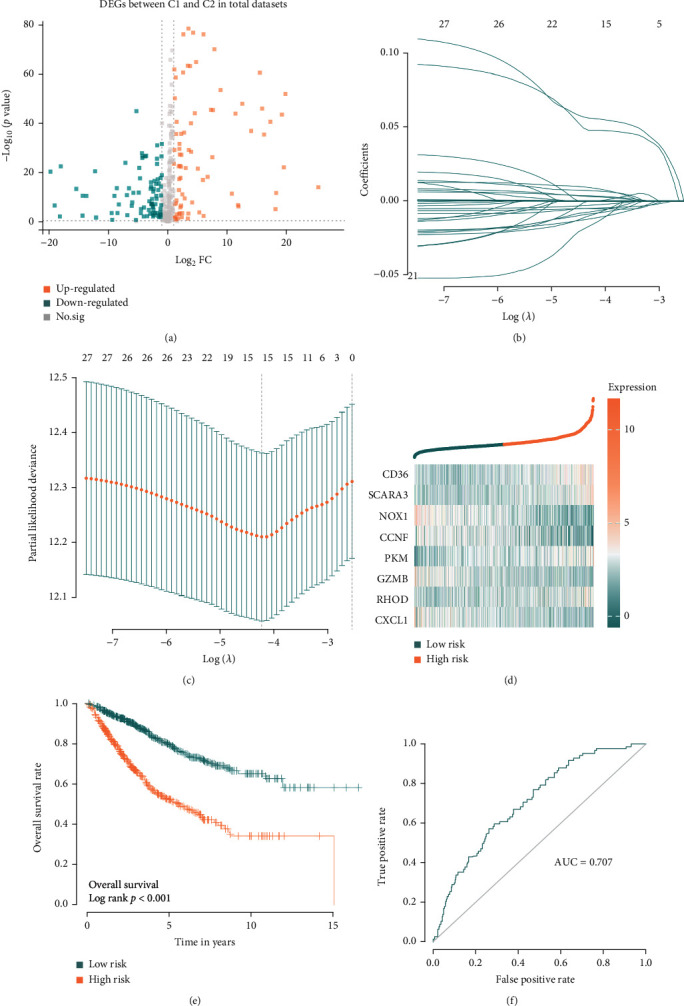
8-gene signature model to predict CRC patients' outcome. DEGs between C1 and C2 in total datasets (a), Lasso regression discover 15 genes that are important (b&c), after multivariate Cox and eight model genes are selected and eight genes signature are built (d). In this model, patients with low-risk score always mean a better outcome, when compared with high-risk score patients (e), and the ROC demonstrates that this model has a good predictive ability for patients' prognosis (f).

DEGs between two subtypes are selected and input into batch univariate Cox regression to screen prognosis genes. To identify more accurate prognosis-related genes, we use strict criteria as a *p* value less than 0.01. Finally, we obtain 27 prognosis-related genes (Supplement Table 1). These 27 genes for future study to continue to perform dimensionality reduction by Lasso regression (LR). The LR analysis results suggest that when lambda obtains the minimum value, 15 important genes are screened (Figures 3(b) and 3(c)). Next step, multivariate Cox regression will analyze these important genes and confirm the final model genes, then, we use the multivariate Cox coefficient and gene expression value to build the final 8 OS-related gene signatures to predict the survival status of CRC patients (Table 1). Each patient will obtain one risk score after inputting 8 genes expression into the model, and the risk curve and genes expression heat map is shown in Figure 3(d). Patients with a risk score of more than the median value will be defined as high-risk groups while others will become low-risk groups. Survival analysis results show that the low-risk group always means a better outcome, while the high-risk group with a poor overall survival rate (log-rank *p* < 0.001) (Figure 3(e)). Furthermore, The ROC results, AUC = 0.707, demonstrate that these 8 OS-related gene signatures also have better predictive performance (Figure 3(f)).

### 3.4. Different Expressions of Genes between Two Subtypes in each Dataset

**Figure 4:**
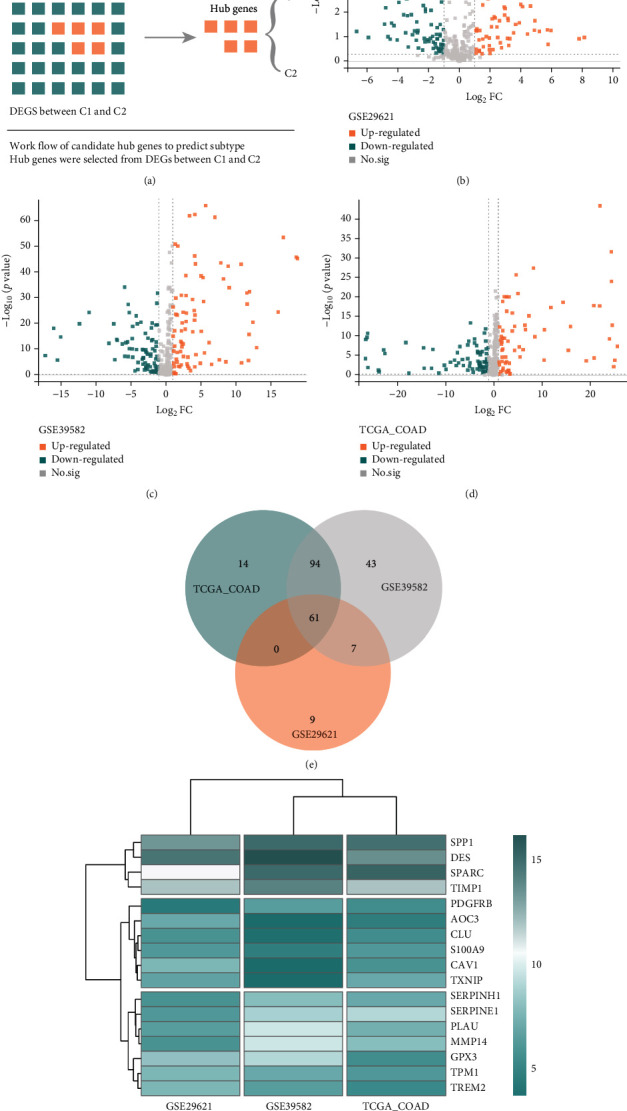
DEGs and RRA analysis to select important genes. The workflow of hub genes to predict subtypes (a), and different expression genes in each datasets (b–d) merge different DEGs (e) and use RRA to select hub genes (f).

The workflow of how to select candidate hub genes, which will be performed to predict OS-related subtypes of CRC patients, is shown in Figure 3(a). In this workflow, we could find that DEGs analysis is the main idea to select hub genes (Figure 4(a)). So, in the next step, we conduct this process, and the different expressions analysis between C1 and C2 in each dataset show that a total of 77 DEGs in GSE29621, including 55 upregulated DEGs and 22 downregulated DEGs; while 205 DEGs in GSE39582, including 111 upregulated DEGs and 94 downregulated DEGs; 169 DEGs in COAD, including 90 upregulated DEGs and 79 downregulated DEGs, respectively (Figures 4(b)–4(d)). In addition, Kobas results show that these DEGs are enrichment in cellular response to oxidative stress, positive regulation of inflammatory response, HIF-1 signaling pathway, and metabolic pathways (Supplement Table 2 and 3).

### 3.5. Fifteen Important DEGs in Different DEGs Expression Profile

**Figure 5:**
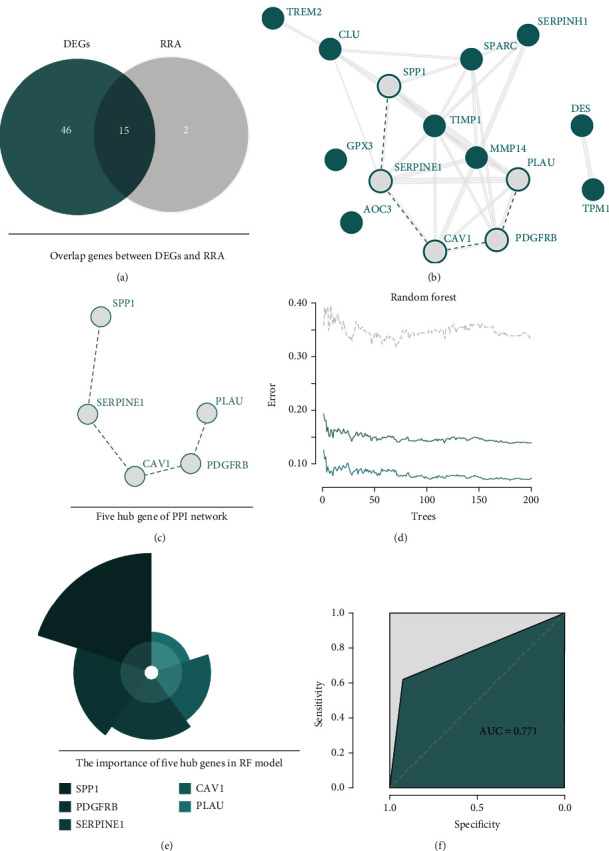
Five hub genes predict two oxidative stress subtypes by random forest. The merge cogenes of DEGs and RRA results (a) and to perform PPI network analysis to select hub genes (b). Five hub genes are identified (c) and could predict patients subtype with high accuracy by random forest algorithm (d–f).

We merge the above results of different expressions of genes in each dataset, and the results show that 61 genes overlap these three datasets (Figure 4(e)), and the RRA analysis shows that 17 genes are important genes in these three datasets (Figure 4(f)). Merge 61 genes and RRA results, we could find that 15 genes are shared genes (Figure 5(a)).

### 3.6. Five Hub Genes Identify

We input fifteen important DEGs into the string database to construct the PPI network, and this network shows that excluding GPX3, AOC3, DES, and TPM1, the other 11 proteins interact closely (Figure 5(b)). In addition, we further apply MCODE plugins in these 11 proteins, and the hub protein network was extracted. The five hub genes that construct this hub network also were identified. They are SPP1, SERPINE1, CAV1, PDGFRB, and PLAU (Figure 5(c)).

### 3.7. Prediction Model of OS-Related Subtype of CRC

**Figure 6:**
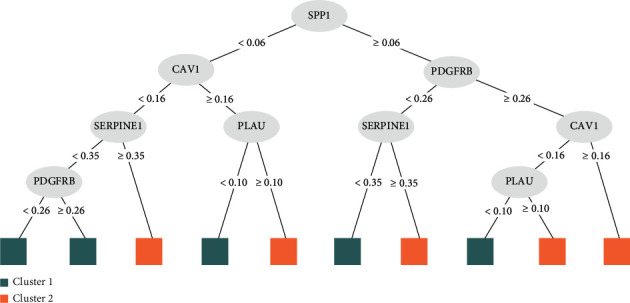
Decision tree of five hub genes prediction model. The decision tree of predict procedure, when we input patients genes expression value, the model will follow the value cut off to make decision, and step by step, at the end, return a subtype results C1 or C2 to assistant clinical decision.

Five hub genes are used to build a prediction model of OS-related subtype of CRC by random forest, and the best mtry value is 2 while the number of trees is 200 in the training set when the model obtains robust predictability (Figure 5(d)).  Figure 5(e) shows the importance of five hub genes. In addition, the high effective ability of the prediction model is also demonstrated by test data. The model has a high AUC value, 0.771, in the test group (Figure 5(f)). We also show the decision tree of the model (Figure 6). According to this decision tree, clinic physicians could evaluate patients' OS-related subtypes by judging five hub genes expression, step by step.

## 4. Discussion

The regulation of OS is an important factor in tumor development. OS not only induces the formation of tumors by abnormal cell proliferation [16] but also promotes further tumor development by altering the metabolism of tumors [17, 18]. Studies have also shown that targeting the antioxidant capacity of tumor cells can have a positive impact on cancer therapy [19]. OS is associated with colorectal carcinogenesis and has been identified as an important risk factor for colorectal adenoma in several studies [20–23]. So, focusing on the role of OS-related genes in CRC is necessary to promote diagnostic gene screening and therapeutic strategies for CRC. Moreover, the small size of the dataset will reduce the accuracy of the predictions and the robustness of the subtypes. Given these, we utilized 3 datasets from the TCGA and GEO databases to identify robust subtypes of CRC for better understanding the underlying molecular pathogenesis of CRC. NMF algorithm has been widely applied to reveal various cancer subtypes through clustering tumor samples [24]. In our study, based on OS-related DEGs, we successfully classified the CRC sample into two subtypes (C1 and C2) by using the NMF algorithm. The results of the PCA analysis revealed that our classification was robust. Then, survival analysis results indicated that C1 subtype had a better prognosis compared to C2 subtype.

It is well known that the prognosis of cancer affects clinical decision-making. Recent clinical guidelines have emphasized the importance of using multigene tests to select patients who should receive adjuvant therapy [25]. Multiple genes of the tests are called cancer signatures, which are crucial for cancer prognosis. Considering the impact of OS and OS subtypes on clinical outcomes in CRC patients, therefore, univariate Cox regression, Lasso Cox regression, and multivariate Cox regression analyses were conducted to construct the optimal OS-related prognostic signature based on DEGs between C1 and C2 subtypes. In this 8-gene prognostic model, as expected, patients in the low-risk group had a better overall survival rate. At the same time, our results showed that this signature had good predictive accuracy in predicting the overall survival of CRC patients. Overall, the prognostic model we constructed may be useful for clinical treatment and decision-making in CRC.

Through machine learning algorithms and the PPI network, we successfully identified five OS-related hub genes, including SPP1, PDGFRB, SERPINE1, CAV1, and PLAU. All five hub genes play a significant role in tumor progression, invasion, and metastasis. Among these genes, secreted phosphoprotein 1 (SPP1, also known as osteopontin) is a secreted glycophosphoprotein, which can be secreted by a variety of cells, including macrophages and endothelial cells [26]. Previous works have demonstrated that SPP1 is overexpressed in various cancers (such as nonsmall cell lung cancer [27] and ovarian cancer [28]) and involved in the progression and metastasis of cancer. In colorectal cancer, SPP1 expression was significantly upregulated, and it promoted CRC metastasis by activating the epithelial-mesenchymal-transition pathway [29]. Platelet-derived growth factor receptor type *β* (PDGFRB, also called PDGFR*β*) has been identified as a causal gene for idiopathic basal ganglia calcification [30]. Meanwhile, it is also correlated with CRC invasion and metastasis, for example, excessive PDGFR*β* signaling leads to oversecretion of THBS4 and proliferative colorectal tumor development [31]. As for the serpin peptidase inhibitor, clade E, member 1 (SERPINE1, also called), it is expressed in many cancer cell and regulates cancer growth, invasion, and angiogenesis [32]. In the CRC cell line, the study has demonstrated that SERPINE1 expression is increased and related to tumor invasiveness and aggressiveness [33]. And, PAI-1 is regarded as a biomarker of poor prognosis in various human cancers and a possible therapeutic target for some cancers [34]. Caveolin-1 (CAV1), an oncogenic membrane protein related to endocytosis, extracellular matrix organization, cholesterol distribution, cell migration, and signaling has been linked to several cancers [35, 36]. For instance, Yang et al. [37] have reported that overexpression of CAV1 markedly inhibits the proliferation, migration, and invasive potential of CRC cells, possibly by reducing phosphorylation of epidermal growth factor receptor activation. Lastly, plasminogen activator (PLAU) is also associated with the complex phenotype of human cancer, and its upregulation promotes metastatic cancers [38]. In a CRC study, downregulation of PLAU expression inhibits CRC cell proliferation and progression [39].

Although we have stratified OS molecular subtypes, built OS-related prognostic model and identified hub genes in CRC for the first time, there are some shortcomings in the current study. First, since we used different platform data and multiple CRC tissue samples, the effect of batch correction may not be completely eliminated. Second, the OS-related prognostic signature constructed from public datasets of the TCGA and GEO databases should be validated for their prognostic value through large-scale prospective studies. Finally, the function of hub genes and their mechanisms affecting CRC development also need to be further elucidated.

## 5. Conclusion

Our study successfully utilizes multiple datasets to stratify CRC samples into two novel OS subtypes, which can provide new insights into the molecular features of CRC. The OS-related prognostic gene signatures can serve as a powerful tool for overall survival prediction and treatment guidance in CRC patients. Additionally, we identified five key genes (SPP1, PDGFRB, SERPINE1, CAV1, and PLAU) as potential biomarkers for predicting the OS subtype and diagnosis of CRC. In general, these findings might enhance our understanding of the molecular pathogenesis of CRC and contribute to identifying new candidate biomarkers for CRC.

## Figures and Tables

**Table 1:** Eight OS-related prognostic genes for signature model by multivariate Cox regression model.

Gene	Coef	HR	HR.95L	HR.95H	*p* value
CD36	0.070893982	1.073467413	1.008614396	1.142490423	0.025764595
SCARA3	0.061080412	1.062984388	0.992720726	1.138221233	0.080019034
NOX1	-0.007467179	0.992560631	0.987524046	0.997622904	0.004016322
CCNF	-0.079307037	0.923756253	0.855402943	0.997571521	0.043181104
PKM	0.005245057	1.005258837	1.002050265	1.008477682	0.001301438
GZMB	-0.019493712	0.980695062	0.964974318	0.996671918	0.018065092
RHOD	0.016390642	1.016525706	1.000464261	1.032845001	0.043686726
CXCL1	-0.005312535	0.994701551	0.98998481	0.999440765	0.028478596

## Data Availability

The datasets were obtained from the TCGA (colon adenocarcinoma: COAD) and GEO (GSE39582 and GSE29621) databases, respectively. Oxidative stress (OS) related genes were checked by the GeneCards database (https://www.genecards.org/).

## Supplementary Materials

Supplementary 1 Supplement Table 1: batch univariate Cox regression discovers twenty-seven prognosis genes in DEGs between two subtypes.

Supplementary 2 Supplement Table 2: Gene Oncology enrichment results of DEGs between C1 and C2 cluster.

Supplementary 3 Supplement Table 3: KEGG enrichment results of DEGs between C1 and C2 cluster.

Supplementary 4 Supplement Figure 1: the heat map for two OS-related subtypes.
