# Supplementary figures and images for: Machine Learning Predicts the Oxidative Stress Subtypes Provide an Innovative Insight into Colorectal Cancer
Source: Oxid Med Cell Longev. 2023 Apr 21;2023:1737501. doi: 10.1155/2023/1737501 (PMC10147531; doi:10.1155/2023/1737501)

**Supplement Figure1.** The heatmap for two OS-related subtypes.

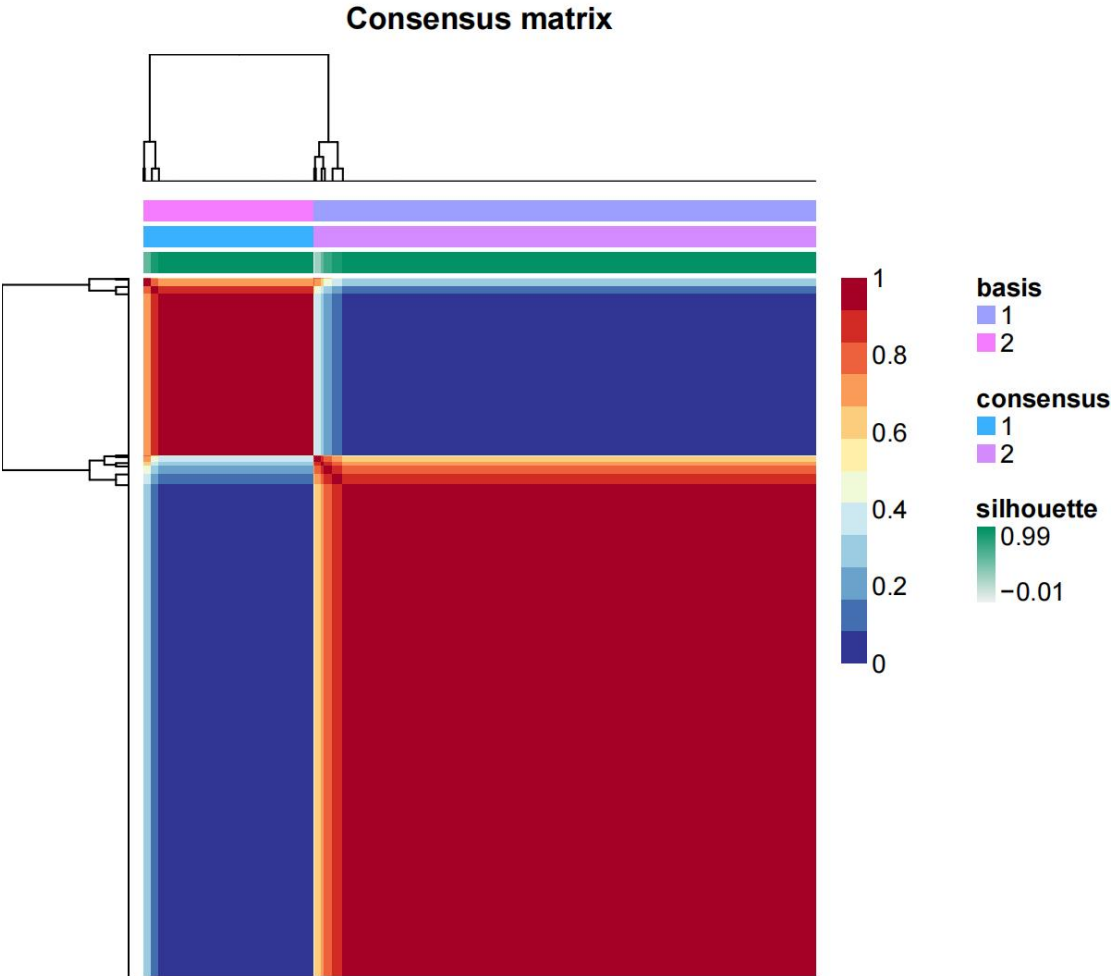

Supplement: Supplementary 4 — Supplement Figure 1: the heat map for two OS-related subtypes. [file 1737501.f4.pdf]
